# Polymorphic variants *INSIG2* rs6726538, *HLA* *‐* *DRB1* rs9272143, and *GCNT1P5* rs7780883 contribute to the susceptibility of cervical cancer in the Bangladeshi women

**DOI:** 10.1002/cam4.3782

**Published:** 2021-02-14

**Authors:** Md. Emtiaz Hasan, Maliha Matin, Md. Enamul Haque, Md. Abdul Aziz, Md. Shalahuddin Millat, Mohammad Sarowar Uddin, Md. Mizanur Rahman Moghal, Mohammad Safiqul Islam

**Affiliations:** ^1^ Department of Pharmacy Noakhali Science and Technology University Noakhali Bangladesh; ^2^ Department of Pharmacy Mawlana Bhashani Science and Technology University Tangail Bangladesh

**Keywords:** cervical cancer, *GCNT1P5*, *HLA‐DQB1*, *HLA‐DRB1*, *INSIG2*, T‐ARMS‐PCR

## Abstract

**Objective:**

Cervical cancer is a gynecological health problem, affecting nearly 500,000 women each year worldwide. Genome‐wide association studies have revealed multiple susceptible genes and their polymorphisms for cervical carcinoma risk. We have carried out this case‐control study to investigate the association of *INSIG2* rs6726538 (A; T), *HLA*‐*DRB1* rs9272143 (T; C), and *GCNT1P5* rs7780883 (G; A) with cervical cancer.

**Methods:**

The present study recruited 234 cervical cancer patients as cases and 212 healthy females as controls. We have applied the tetra‐primer amplification refractory mutation system polymerase chain reaction (T‐ARMS‐PCR) method for genotyping.

**Results:**

The SNP rs6726538 was significantly associated with increased risk of cervical cancer in all genetic models (AT vs. AA: OR = 3.30, 95% CI = 2.19–4.97, *p* < 0.0001; TT vs. AA: OR = 8.72, 95% CI = 3.87–19.7, *p* < 0.0001; AT+TT vs. AA: OR = 3.87, 95% CI = 2.61–5.73, *p* < 0.0001; T vs. A: OR = 2.97, 95% CI = 2.20–4.01, *p* < 0.0001) except the recessive model which showed a significantly reduced risk (TT vs. AA+AT: OR = 0.20, 95% CI = 0.09–0.44, *p* = 0.0001). rs9272143 showed significantly reduced risk for the additive model 1, dominant model, and allelic model (TC vs. TT: OR = 0.46, 95% CI = 0.31–0.70, *p* = 0.0004; TC+CC vs. TT: OR = 0.47 95% CI = 0.32–0.70, *p* = 0.0002; C vs. T: OR = 0.56, 95% CI = 0.40–0.78, *p* = 0.0006, respectively). The third variant, rs7780883, was significantly associated with increased risk in additive model 2, dominant, and allelic models (AA vs. GG: OR = 5.08, 95% CI = 2.45–10.5, *p* < 0.0001; GA+AA vs. GG: OR = 1.54, 95% CI = 1.06–2.24, *p* = 0.0237; A vs. G: OR = 1.88, 95% CI = 1.34–2.52, *p* < 0.0001, consecutively), whereas recessive model reduced the risk of cervical cancer (AA vs. GG+GA: OR = 0.20, 95% CI = 0.09–0.41, *p* < 0.0001). Other models of these SNPs were not associated with cervical cancer. All significant associations for three SNPs withstand after Bonferroni correction except the additive model 2 of rs7780883.

**Conclusion:**

Our study concludes that *INSIG2* rs6726538, *HLA*‐*DRB1* rs9272143, and *GCNT1P5* rs7780883 polymorphisms may contribute to the development of cervical cancer in the Bangladeshi population.

## Graphical abstract

Cervical cancer (CC) is a gynecological health problem, affecting nearly 500,000 women each year worldwide. The present study recruited 234 cervical cancer patients as cases and 212 healthy females as controls. Our study concludes that rs6726538, rs9272143, and rs7780883 polymorphisms may contribute to the development of CC in the Bangladeshi population.

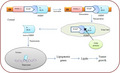

## 1 INTRODUCTION

Cervical cancer (CC) is the world's fourth most common type of female malignancy that represents a burden on global health considering incidence and mortality. CC constitutes almost 6.6% of all cancers in women, and around 570,000 new cases were recorded in 2018. CC is the second leading cause of death from cancer in women between the ages of 20 and 39, causing nine deaths per week in this age group.^1^, ^2^, ^3^ In addition, it is the second most frequent cause of death among women aged 15 to 44 years old.^4^ In Bangladesh, cervical cancer is the second most common cancer in women, with a total of 11,956 women diagnosed each year in Bangladesh, and over 6582 of them die each year.^5^, ^6^

Human papillomavirus (HPV) is thought of as one of the main precursors of cervical cancer induced by persistent infection.^7^, ^8^ Despite the availability of some vaccines those work against some different types of HPV, reducing the risk of developing cervical cancer,^1^ individuals’ genetic susceptibility to the disease may control the immune response.^9^ During the early stages, HPV‐associated cervical cancer progresses asymptomatically. In most cases, the virus may remain undetected if not tested in time and starts tumor formation, leading to cervical cancer development.^10^ Screening within the ages of 30 to 49 years may prevent the development, and follow‐up diagnosis and treatment increase the possibility to remove cancer in the early stage.^1^ However, research has demonstrated that HPV alone does not entirely clarify cervical malignancy; instead, various cofactors are related to the progression of this carcinogenicity.^11^ Additional host factors, such as women's age, number of abortions, first delivery age, multiple childbirths, multiple sexual partners, oral contraceptive pills, immune suppression, smoking of cigarettes, and poor socioeconomic condition, are also responsible for the development of CC. Moreover, multiple genetic factors play a significant role in the progression of developing cervical carcinoma.^12^, ^13^, ^14^

*INSIG2* (insulin‐induced gene 2) is an endoplasmic reticulum (ER) protein that regulates or blocks the proteolytic activation of sterol regulatory element‐binding proteins (SREBPs), transcription factors that may trigger fatty acid and cholesterol synthesis. Studies explicated that *INSIG2* was associated with obesity.^15^, ^16^, ^17^, ^18^ It was also reported to be a biomarker for the development of colon cancer ^17^ and pancreatic cancer.^19^ This evidence suggests that *INSIG2* might show an emergent contribution to the progression of cancers. Howsoever, no previous studies are suggesting the involvement of *INSIG2* in cervical cancer except a GWAS study.^20^ Intergenic rs6726538 (A; T) is located on chromosome 2 (2q14.2), and the A allele of rs6726538 is documented to be associated with the progression of cervical cancer (OR = 1.95, 95% CI = 1.47–2.57 and *p* = 2.76 × 10^−6^ for the risk allele A).^20^

SNP rs9272143 (T; C) is located in the class II region (major histocompatibility complex, MHC region) of chromosome 6 (p‐arm, 6p21.32) between *HLA*‐*DRB1* and *HLA*‐*DQB1* and associated with cervical cancer.^21^ More specifically, rs9272143 is positioned at 43.19 kb upstream of *HLA*‐*DRB1* and 4.38 kb upstream of *HLA*‐*DQA1*. *HLA*‐*DRB1* is a member of the HLA class II β‐chain paralogs that encodes the β‐chain of the *HLA*‐*DR*, which is a peptide‐antigen receptor. It is commonly found in the antigen‐presenting cells (APCs) of squamous epithelia in the cervix and Langerhans cells (LC). It was observed that *HLA*‐*DRB1* plays a major role in the cell‐mediated immune response by presenting antigens to CD4^+^ helper T cell, which after activation, secretes different small proteins or cytokines.^22^, ^23^, ^24^ We have tried to keep the focus on the rs9272143 variant concerning the progression of cervical cancer as *HLA*‐*DRB1* expression is found in the cervix.

rs7780883 (G; A) SNP of glucosaminyl (N‐acetyl) transferase 1 pseudogene 5 (*GCNT1P5*) is located on an intergenic region of chromosome 7, and a genome‐wide association study (GWAS) revealed that the A allele of rs7780883 is associated with the development of cervical cancer (OR = 3.28, 95% CI = 1.97–5.5 and *p* = 2.49 × 10^−6^ for the risk allele A). Therefore, we have tried to find out the risk susceptibility of this variant with cervical carcinogenesis.^20^

GWAS with these three different novel variants (rs6726538 of *INSIG2*, rs9272143 of *HLA*‐*DRB1*, and rs7780883 of *GCNT1P5*) imposed the probability of cervical cancer progression. Again, due to geographical or ethnic differences, the rate and extent of disease occurrences in different world regions are observed. Even the variation can be found in the same ethnicity.^25^ So, we tried to find out the association of cervical cancer with these SNPs in patients from various regions of Bangladesh. To add, we choose the tetra‐primer amplification refractory mutation system–polymerase chain reaction (T‐ARMS–PCR) method to perform the study that works with simple gel electrophoresis following the PCR run.^26^

## 2 MATERIALS AND METHODS

### 2.1 Study design and sample recruitment

We carried out the present case‐control study in 234 cervical cancer patients recruited as cases from the National Institute of Cancer Research and Hospital, Dhaka, Bangladesh. Two hundred and twelve healthy volunteers were recruited as controls from the different parts of the country. Histologically diagnosed cervical cancer was confirmed in the patients, and controls were chosen by matching age with the cases. The exclusion criteria for both the case and the controls are: (a) those under the age of 21; (b) those unable to provide relevant data; (c) those suffering from comorbidities or chronic diseases; and (d) those who declined to participate in the study. All participants signed and agreed to participate in this study, also consenting to the consequent publication of the results, according to the written consent form. In terms of lifestyle and sociodemographic factors associated with an increased risk of cervical cancer, each patient and control subjects were interviewed. The tumor location, tumor stage, histological type, and condition of lymph nodes were collected from the patients’ medical records. The study protocol and consent form were reviewed and approved by the ethics committee of the National Institute of Cancer Research and Hospital, Bangladesh (NICRH/Ethics/2019/447). The research was carried out following the principles of conducting research on human subjects according to the Helsinki Declaration and its further correction.^27^ This study was conducted at the Pharmacogenomics and Molecular Biology Lab, Department of Pharmacy, Noakhali Science and Technology University, Bangladesh.

### 2.2 Collection and storage of blood, isolation, and quantification of genomic DNA

Peripheral blood (around 3 ml) was collected from individuals from both the groups and transferred to a plastic tube containing EDTA (ethylenediaminetetraacetic acid) and stored at −80°C until the extraction of DNA. Genomic DNA was isolated using “FavorPrep” DNA extraction mini kit following the protocol book supplied with the kit. The concentration of extracted DNA was measured using a microvolume spectrophotometer (Genova Nano, Jenway). The absorption ratio of 260 nm and 280 nm was used to assess the genomic DNA purity.

### 2.3 Primer design and genotyping

Genotyping of SNPs was completed employing the tetra‐primer amplification refractory mutation system‐polymerase chain reaction (ARMS‐PCR) method.^28^ A few online programing were utilized for primer design. Four primers, such as forward outer, reverse outer, forward inner, and reverse inner, were designed to amplify the desired allele (Table 1). PCR premix was formulated by adding EmeraldAmp GT PCR Master Mix, nuclease‐free water, MgCl_2_, primers at a specified concentration. For instance, the volume of outer primers (forward outer and reverse outer) was 1.5 μl, and inner primers (forward inner and reverse inner) was 2.5 μl for a 120 μl PCR master mix solution (12 samples; 10 μl/reaction). The PCR was then performed by adding DNA sample with the premix (10 μl) at the required concentration. Next, the PCR products were analyzed by gel electrophoresis (1% agarose gel) to get the confirmation of the DNA bands of specific alleles staining with ethidium bromide.

**TABLE 1:** Primers used in the tetra‐primer ARMS‐PCR method

SNPs	Primers	Sequence (5́−3́)	Allele	Amplicon size (bp)
rs6726538	FI	CAACCCCATCCCCCTTGCTATTTATT	A	261
	RI	GAACACTGATTATGTGATAGTCTTCCTGAT		231
	FO	GAGTAGCTGGGACTACAAGCACACACTA	T	432
	RO	CTCACTTTCCACAAACTTTGAAGGAAGA		432
rs9272143	FI	CATAAAAAAATCTGACAGATACAAGCGC	T	222
	RI	ATTGCTGAAAAACAAAATTTTTTTGACGA		177
	FO	ACCTATTGATGCTACAGAGATGTGAGGG	C	342
	RO	AATGATAATAACATCATGCTTTGGGCTG		342
rs7780883	FI	TACACTCTGAAGCTGTGACTACTGGCA	G	143
	RI	TTTGTATCCAAAGGGTATACTGGATACAC		201
	FO	ATAAAACATGTCAAAATTAAGGAAGGGG	A	288
	RO	TGTATATTTCTTTGGGCATAGTCCATCT		288

Abbreviations: FI, Forward inner; FO, Forward outer; RI, Reverse inner; RO, Reverse outer.

### 2.4 Validation process of tetra‐primer ARMS–PCR method

**FIGURE 1:**
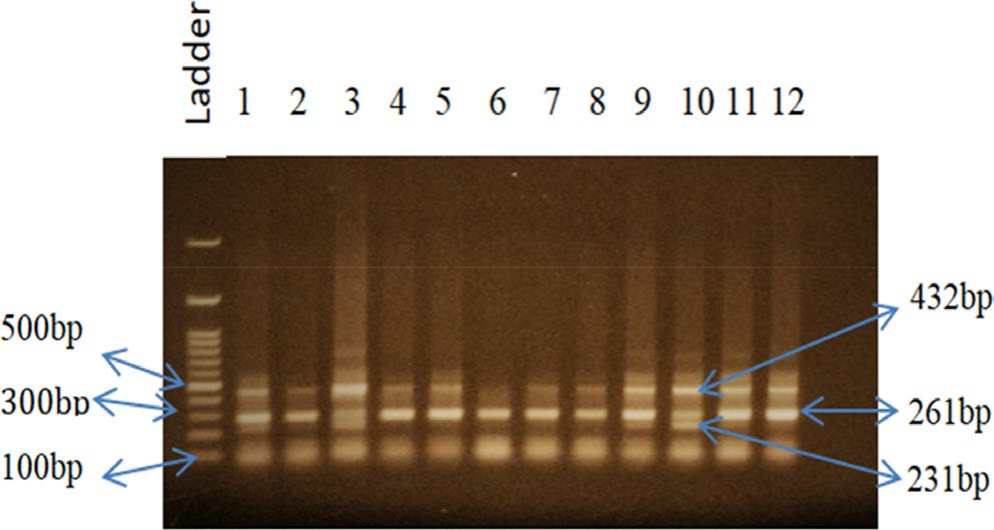
PCR amplification bands for SNP rs6726538 at 64°C annealing temperature

**FIGURE 2:**
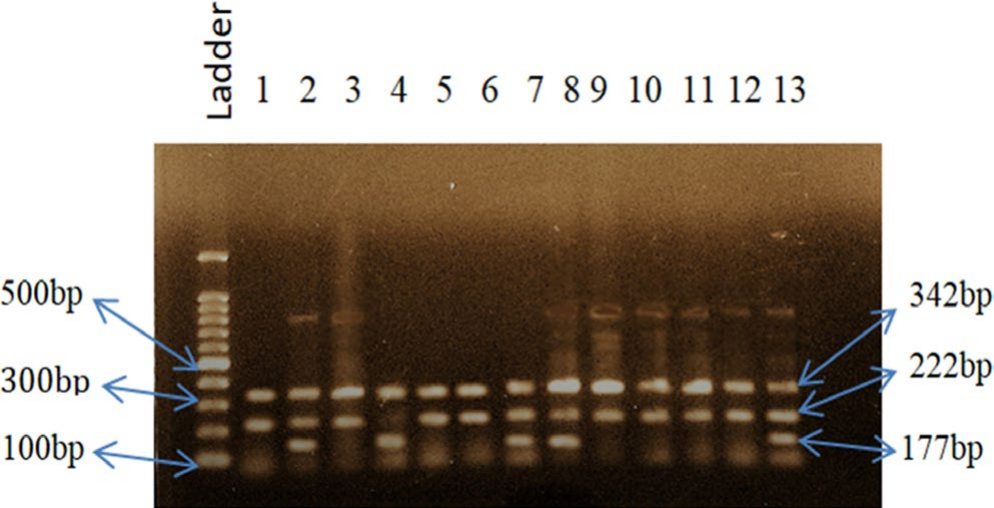
PCR amplification bands for SNP rs9272143 at 51°C annealing temperature

**FIGURE 3:**
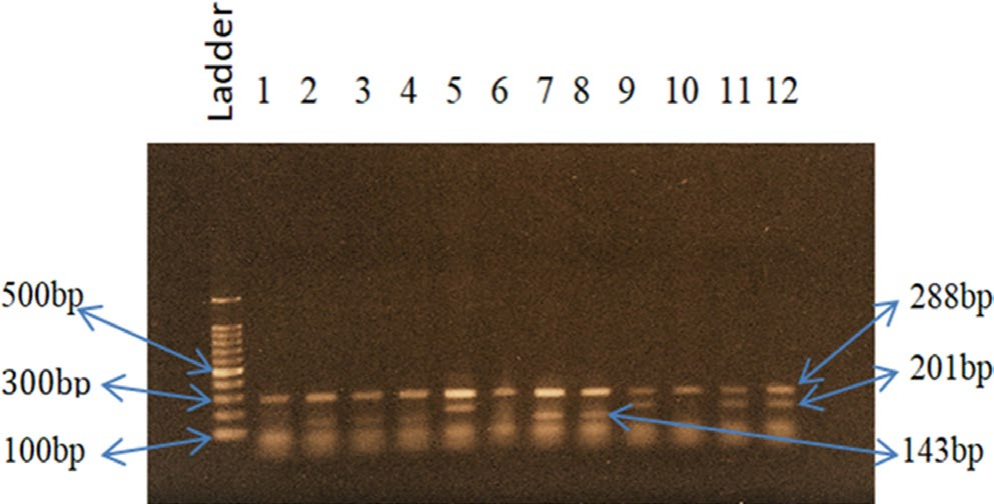
PCR amplification bands for SNP for rs7780883 at 64°C annealing temperature

Method validation was performed for selecting a suitable annealing temperature for the selected SNPs to get the desired DNA fragments. The melting temperatures of primers were calculated manually. We selected 64°C, 51°C, and 64°C temperatures consecutively as our desired annealing temperature for SNPs *INSIG2* rs6726538, *HLA*‐*DRB1* rs9272143, and *GCNT1P5* rs7780883. For rs6726538 SNP, at 64°C temperature, we detected 432 bp, 261 bp, and 231 bp size fragments (Figure 1), whereas for rs9272143 SNP, at 51°C temperature, we detected 342 bp, 222 bp, and 177 bp size fragments (Figure 2) and for rs7780883 SNP, at 64°C temperature, we detected 288 bp, 201 bp, and 143 bp size fragments (Figure 3). The details of PCR conditions for rs6726538, rs9272143, and rs7780883 SNPs with fragment size are illustrated in Table 2.

**TABLE 2:** PCR conditions for rs6726538, rs9272143, and rs7780883 SNPs with fragment size

SNP	PCR conditions	No. of cycles	Size of PCR products (bp)	Genotype
rs6726538	95°C for 5 min		NH: 261,432	AA
	95°C for 1 min		HE: 231,261,432	AT
	64°C for 30 s	35 cycles	MH: 231,432	TT
	72°C for 30 s			
	72°C for 10 min			
rs9272143	95°C for 5 min			
	95°C for 1 min		NH: 222,342	TT
	51°C for 30 s	35 cycles	HE: 177,222,342	TC
	72°C for 1 min		MH: 177,342	CC
	72°C for 10 min			
rs7780883	95°C for 5 min			
	95°C for 1 min		NH: 143,288	GG
	64°C for 30 s	35 cycles	HE: 143,201,288	GA
	72°C for 1 min		MH: 201,288	AA
	72°C for 10 min			

Abbreviations: HE, Heterozygote; MH, Mutant Homozygote; NH, Normal Homozygote.

### 2.5 Statistical calculation

SPSS software package, version 23.0 (IBM, Armonk, NY, USA) was applied for calculating all statistical data. Chi‐square test (χ^2^), odds ratio (OR), and their 95% confidence intervals (CI) and Hardy–Weinberg Equilibrium (HWE) were calculated. The genotype and allelic frequencies were reported as the percentage. For all analyses, a statistically significant value was considered at *p* < 0.05. Bonferroni correction was performed to correct the *p*‐values, and a *p*‐value of <0.0033 (for three SNPs and five genetic models for each SNP, *p* = 0.05/5×3=) after the Bonferroni correction was considered statistically significant.^29^

## 3 RESULTS

### 3.1 Distribution of demographic data between subjects

The demographic characteristics of patients and control subjects are reported in Table 3. The minimum and maximum ages of patients were 35 and 80 years (mean age 57.5 years), and healthy controls were 30 and 75 years (mean age 52.5 years), respectively. The frequencies of patients under 45 years and between 45 and 60 years were 33.33% and 58.1%, respectively, whereas for controls, these were 32.55% and 62.73%, consecutively. The percentage of both patients and controls aged over 60 years was 8.5% and 4.72%, respectively. Besides, most of the patients suffered from stage IIB (38.57%) of cervical cancer. The percentage of stage IIIB cervical carcinoma patients was also high, comprising of 24.29%. The frequency of grade II tumor was higher (42.73%) compared to grades I and III. The negative lymph nodes found in the patients were higher than the positive (90.6% vs. 9.4%) lymph nodes. However, no mentionable data were found for smoking status and alcohol consumption in the patients.

**TABLE 3:** Demographic characteristics of the patients and controls

Variables	Cases, *n* = 234 (%)	Controls, *n* = 212 (%)
Age (Years)		
<45	78 (33.33%)	69 (32.55%)
45–60	136 (58.1%)	133 (62.73%)
>60	20 (8.57%)	10 (4.72%)
Tumor Grade		
I	80 (34.19)	N/A
II	100 (42.73)	N/A
III	54 (23.08)	N/A
Histological Type		
SQC	108 (46.15)	N/A
Adenocarcinoma	53 (22.65)	N/A
SCC	10 (4.27)	N/A
Endometrioid	21 (8.97)	N/A
Other	42 (17.95)	N/A
Tumor Stage		
IIB1‐IIB2	26 (10.95%)	N/A
IIA	9 (3.81%)	N/A
IIB	90 (38.57%)	N/A
IIIA	33 (14.29%)	N/A
IIIB	57 (24.29%)	N/A
IVA‐IVB	19 (8.095%)	N/A
Lymph Nodes		
Negative	212 (90.6)	N/A
Positive	22 (9.4)	N/A
Smoking status	Not found	N/A
Alcohol consumption	Not found	N/A

Abbreviations: SCC, Serous cystadenocarcinoma; SQC, Squamous cell carcinoma.

### 3.2 Genotype distribution and contribution of variants to cervical cancer risk

The genotypic and allelic frequencies and the association of different genetic models of *INSIG2* rs6726538, *HLA*‐*DRB1* rs9272143, and *GCNT1P5* rs7780883 with Bonferroni correction are illustrated in Table 4.

**TABLE 4:** Genotypic distributions and the association of rs6726538, rs9272143, and rs7780883 alleles with cervical cancer risk

SNPs			Cases	%	Controls	%	Genetic models	OR	95% CI	*p*‐value
rs6726538	Genotype	AA	74	31.62	136	64.15	Additive model 1 (AT vs. AA)	3.30	2.19–4.97	**<0.0001** *
							Additive model 2 (TT vs. AA)	8.72	3.87–19.7	**<0.0001** *
		AT	122	52.14	68	32.08	Dominant model (AT+TT vs. AA)	3.87	2.61–5.73	**<0.0001** *
		TT	38	16.24	8	3.78	Recessive model (TT vs. AA+AT)	0.20	0.09–0.44	**0.0001** *
	Allele	A	270	57.70	340	80.19		1		
		T	198	42.31	84	19.81		2.97	2.20–4.01	**<0.0001** *
rs9272143	Genotype	TT	170	72.65	118	55.66	Additive model 1 (TC vs. TT)	0.46	0.31–0.70	**0.0004** *
							Additive model 2 (CC vs. TT)	0.52	0.18–1.54	0.0899
		TC	58	24.79	86	40.56	Dominant model (TC+CC vs. TT)	0.47	0.32–0.70	**0.0002** *
		CC	6	2.56	8	3.77	Recessive model (CC vs. TT+TC)	1.49	0.51–4.37	0.47
	Allele	T	398	85.78	322	75.94		1		
		C	70	15.09	102	24.06		0.56	0.40–0.78	**0.0006** *
rs7780883	Genotype	GG	114	48.72	126	59.43	Additive model 1 (GA vs. GG)	1.08	0.72–1.62	0.7245
							Additive model 2 (AA vs. GG)	5.08	2.45–10.5	**<0.0001** *
		GA	74	31.62	76	35.85	Dominant model (GA+AA vs. GG)	1.54	1.06–2.24	**0.0237**
		AA	46	19.66	10	4.72	Recessive model (AA vs. GG+GA)	0.20	0.09–0.41	**<0.0001** *
	Allele	G	302	64.53	328	77.36		1		
		A	166	35.47	96	22.64		1.88	1.34–2.52	**<0.0001** *

* *p* < 0.05 was considered as statistically significant (bold), whereas * indicates significant after Bonferroni correction (*p* < 0.0033).

With regard to the rs6726538 (A; T) polymorphism in *INSIG2* for the case group, the frequencies of the alleles A and T were 57.7% and 42.31%, respectively, whereas for the control group, the frequencies of the alleles A and T were 80.19% and 19.81%, respectively. The frequency of genotypes AA, AT, and TT in cases were 31.62%, 52.14%, and 16.24%, individually and control frequencies were 64.15%, 32.08%, and 3.78%, consecutively. Women carrying AT genotype showed 3.30 times higher risk for the development of cervical cancer compared to the wild‐type AA, and this result is statistically significant (OR = 3.30, 95% CI = 2.19–4.97, *p* < 0.0001). Like the same way, women carrying TT genotype showed 8.72 times significantly higher risk of developing cervical cancer compared to AA genotype carrying women (OR = 8.72, 95% CI = 3.87–19.7, *p* < 0.0001). Dominant model carriers (women carrying both AT and TT genotypes) had 3.87 times higher risk of developing cervical cancer (OR = 3.87, 95% CI = 2.61–5.73, *p* < 0.0001), and it is statistically significant. Women carrying T allele (minor) also showed 2.97 times higher risk of developing cervical cancer, and the data are statistically significant (T vs. A: OR = 2.97, 95% CI = 2.20–4.01, *p* < 0.0001). Only the recessive model carriers (TT vs. AA+AT) had a protective effect in comparison to AA+AT genotype, and these data are statistically significant (OR = 0.20, 95% CI = 0.09–0.44, *p* = 0.0001). It was found that all associations were statistically significant after performing Bonferroni correction (*p* < 0.0033).

In case of the *HLA*‐*DRB1* rs9272143 (T; C) variant, the frequencies of TT, TC, and CC genotypes were 72.65%, 24.79%, and 2.56%, consecutively in cases, whereas controls had 55.66%, 40.56%, and 3.77%, respectively. The frequency of minor allele C allele was 15.09% in cases and 24.06% in controls. rs9272143 SNP showed reduced risk association for cervical cancer in additive model 1, dominant model, and allele model, and the associations withstand even after Bonferroni correction (TC vs. TT: OR = 0.46, 95% CI = 0.31–0.70, *p* = 0.0004; TC+CC vs. TT: OR = 0.47 95% CI = 0.32–0.70, *p* = 0.0002; C vs. T: OR = 0.56, 95% CI = 0.40–0.78, *p* = 0.0006). No association of cervical cancer was found with additive model 2 and recessive model (CC vs. TT: OR = 0.52, 95% CI = 0.18–1.54, *p* = 0.0899; recessive model: OR = 1.49, 95% CI = 0.51–4.37, *p* = 0.47).

The third variant rs7780883 (G; A) of *GCNT1P5* exhibited a significant association in three genetic models for the increased risk of cervical cancer in the studied sample (additive model 2, AA vs. GG: OR = 5.08, 95% CI = 2.45–10.5, *p* < 0.0001; dominant model, GA+AA vs. GG: OR = 1.54, 95% CI = 1.06–2.24, *p* = 0.0237; allele model, A vs. G: OR = 1.88, 95% CI = 1.34–2.52, *p* = <0.0001) that was also significant after Bonferroni correction except for the dominant model. Women carrying GA and AA genotypes showed 1.08 times higher risk (GA vs. GG: OR = 1.08, 95% CI = 0.72–1.62, *p* = 0.7245) compared to wild allele GG, and the association was found not to be statistically significant. The recessive model showed a reduced risk for cervical cancer development, and it was statistically significant even after Bonferroni correction (AA vs. GG+GA: OR = 0.20, 95% CI = 0.09–0.41, *p* < 0.0001). The frequencies of A allele (minor) were 35.47% and 22.64% in the cases and controls, respectively. The percentages of GG, GA, and AA genotypes in cases were 48.72%, 31.62%, 19.66%, respectively, and in controls, these values were 59.43%, 35.85%, and 4.72%, consecutively.

## 4 DISCUSSION

Cervical cancer (CC) is responsible for extreme mortality among women throughout the world. Though HPV infection is the major cause of developing cervical cancer, almost 70% to 90% of patients can recover from this infection. However, several host factors are responsible for the high prevalence of this disease, as well as the mortality caused by it. Poor development of the immune system, environmental factors, as well as genetic variations or alterations lead to high susceptibility.^14^, ^30^, ^31^, ^32^, ^33^ GWAS have been carried out to correlate the genetic polymorphisms or variability responsible for the predisposition of cervical cancer in females. Moreover, interpreting the function of genetic polymorphisms in cervical carcinoma leads to the discovery of personalized medicine.^34^ Due to the variable genetic polymorphisms between different ethnic groups, only the successful identification of these polymorphisms and quantification of gene expression can help to manage cancer.^35^, ^36^ Our study found the association of *INSIG2* rs6726538 (A; T), *HLA*‐*DRB1* rs9272143 (T; C), and *GCNT1P5* rs7780883 (G; A) polymorphisms with cervical cancer risk in the Bangladeshi population.

**FIGURE 4:**
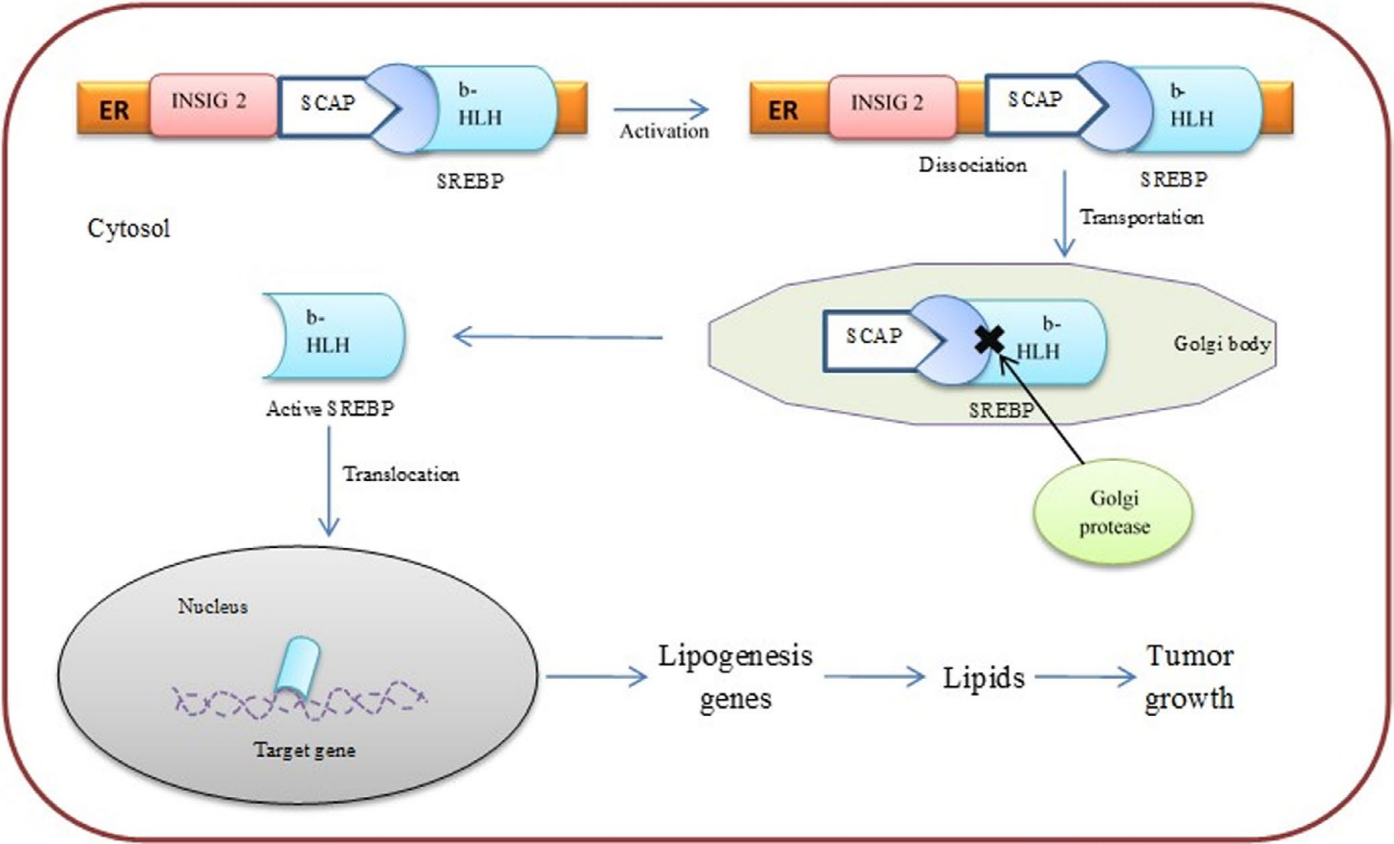
Mechanism of *INSIG2* in cancer. Inactive sterol regulatory element‐binding protein (SREBP) is synthesized in the endoplasmic reticulum (ER) and forms complex with SREBP cleavage‐activating protein (SCAP) and *INSIG2*. When sterol level reduces, complex dissociates and SREBP transports into the Golgi body. After cleavage by proteases, active basic‐helix‐loop‐helix (b‐HLH) is transported into the nucleus

As far as we know, more than 30 susceptible genes have been reported for their crucial role in the progression of cervical malignancy, namely *HLA B*/*C*, tumor necrosis factor‐α (*TNF*‐*α*), interleukin 10 (*IL10*), interleukin 12 (*IL12*), cytochrome P450 1A1 (*CYP1A1*), *p53*, *p16*, *p21*, poly [ADP‐ribose] polymerase 1 (*PARP1*), BRCA1‐interacting protein 1 (*BRIP1*), cytotoxic T lymphocyte antigen 4 (*CTLA4*), X‐ray repair cross‐complementing protein 1 (*XRCC1*), *Caspase 8*, C‐C chemokine receptor type 2 (*CCR2*), exonuclease 1 (*EXO1*), phosphatidylinositol‐4,5‐bisphosphate 3‐kinase catalytic subunit alpha (*PIK3CA*), fas ligand (*FASLG*), fas receptor (*FASR*), HOX transcript antisense RNA (*HOTAIR*), interferon‐γ (*IFN*‐*γ*), murine double minute 2 (*MDM2*), BH3‐interacting domain death agonist (*Bid*), *Tap2TLR9*, and methylenetetrahydrofolate reductase (*MTHFR*).^30^, ^44^ *INSIG2* is an important ER protein that possibly inhibits the processing of SREBPs through binding to the cleavage activating protein (SCAP) in a well‐controlled fashion and preventing the proteolytic mechanism of SREBPs by enzymes of the Golgi apparatus (Figure 4). This inhibitory mechanism leads to the blockage of cholesterol synthesis.^18^, ^45^

The expression of *INSIG2 *has been associated with the progression of colorectal cancer metastasis and its outcome.^17^, ^46^, ^47^ An investigation has explicated the association of *INSIG2* with pancreatic cancer.^19^ This evidence suggests the probability of this gene with cervical cancer. We have investigated and found the association of rs6726538 of *INSIG2* with the risk of cervical cancer in the Bangladeshi population. Our results demonstrated that all the genetic models were associated with cervical malignancy, and the associations were significant even after the Bonferroni correction. The frequency of minor allele T was higher in the patient group (42.31%) than in the controls (19.81%). We have observed that the frequency of AT (52.14%) and TT (16.24%) genotypes in the patient group was also higher than the control group.

GWAS and other genetic association studies have been investigated with the human leukocyte antigen (HLA) loci in cervical neoplasia. Studies have reported that the haplotype *HLA*‐B*0702‐DRB1*1501/HLADQB1*0602 increases the risk of cervical carcinoma, whereas the haplotype *HLA*‐B*1501/*HLA*‐DRB1*1301/*HLA*‐DQA1*0103/*HLA*‐DQB1*0603 protects from the risk of the disease progression.^21^, ^23^, ^24^, ^48^, ^49^, ^50^, ^51^, ^52^ A study of 84 Finnish individuals showed that the rs9272143 variant was a cis‐expression quantitative trait locus (eQTL), which alters the expression of *HLA*‐*DRB1* in fatty tissue, and the C allele significantly increases the expression of *HLA*‐*DRB1*.^24^ Leo et al. also explicated a strong correlation of cervical cancer with both risk and protective HLA haplotypes. These are ascertained by the presence of amino acids of *HLA*‐*DRB1* at positions 13 and 71 in pocket 4 and for *HLA*‐*B* at position 156.^48^ However, on account of rs9272143 polymorphism, we have found that the TC, TC+CC genotype, and C allele act like a protective factor in Bangladeshi cervical cancer patients. These associations were also significant after the Bonferroni correction. We have also reported that the AA mutant homozygote frequency is higher in patients than in controls. The frequency distribution showed a lower frequency of minor allele C in cases compared to healthy controls.

The third variant of our study is rs7780883 SNP in *GCNT1P5* gene, which is an intergenic variant and located on chromosome 7. GWAS have reported this variant as a risk factor for cervical cancer development.^20^ However, to the best of our knowledge, no previous association study or case‐control study was conducted on this polymorphism with the risk of cervical malignancy. Consequently, this is the first case‐control analysis of rs7780883 in the world and in Bangladesh to provide proof of association with cervical cancer, except for a GWAS study. Our study demonstrates that the additive model 2, the dominant model, and the minor allele A increased the risk of cervical cancer significantly, whereas the recessive model significantly reduced cervical cancer risk. All the associations withstand after Bonferroni correction except the dominant model. The frequency of minor allele A was higher (35.47%) in cancer patients than in the healthy controls.

Our present study also found that the patients aged between 45 and 60 years are at higher (58.1%, 95%Cl = 51.50–64.50, *p* < 0.0001) risk than other age groups in Bangladesh. Moreover, the frequency of patients with tumor stages IIB and IIIB were higher than other stages of tumor comprising 38.57% (95%Cl = 32.30–45.13, *p* < 0.0001) and 24.29% (95%Cl = 18.94 to 30.30, *p* < 0.0001), respectively, for the patients. We have also validated tetra‐primer ARMS‐PCR for the genotyping of these variants for the first time.

To mention, besides the strengths of our study findings, it had some lacking as our sample size was not appreciable. In this case, although the sample was somewhat small, we found the association of cervical cancer with all the three SNPs after performing the Bonferroni correction.

## 5 CONCLUSION

Our study summarizes that *INSIG2* rs6726538 (A; T), *HLA*‐*DRB1* rs9272143 (T; C), and *GCNT1P5* rs7780883 (G; A) polymorphisms are associated with cervical cancer development in the Bangladeshi population. We have identified the association of these variants with cervical carcinoma for the first time in Bangladesh. However, we suggest further study on a large scale on different cohorts to find in detail genotype–phenotype associations of these variants.

## ETHICAL DECLARATIONS

The research protocol and consent form were reviewed and approved by the ethics committee of the National Institute of Cancer Research and Hospital (NICRH). The ID of ethical approval was NICRH/Ethics/2019/447.

## Data Availability

The datasets used in this study are available from the corresponding author on a reasonable request.
